# From Emotions to Consciousness – A Neuro-Phenomenal and Neuro-Relational Approach

**DOI:** 10.3389/fpsyg.2012.00303

**Published:** 2012-08-31

**Authors:** Georg Northoff

**Affiliations:** ^1^Mind, Brain Imaging and Neuroethics Research Unit, Institute of Mental Health Research, University of OttawaOttawa, ON, Canada

**Keywords:** consciousness, emotion, emotional feeling, insula, James–Lange theory

## Abstract

The James–Lange theory considers emotional feelings as perceptions of physiological body changes. This approach has recently resurfaced and modified in both neuroscientific and philosophical concepts of embodiment of emotional feelings. In addition to the body, the role of the environment in emotional feeling needs to be considered. I here claim that the environment has not merely an indirect and instrumental, i.e., modulatory role on emotional feelings via the body and its sensorimotor and vegetative functions. Instead, the environment may have a direct and non-instrumental, i.e., constitutional role in emotional feelings. This implies that the environment itself is constitutive of emotional feeling rather than the bodily representation of the environment. I call this the relational concept of emotional feeling. The present paper discusses recent data from neuroimaging that investigate emotions in relation to interoceptive processing and the brain’s intrinsic activity. These data show the intrinsic linkage of interoceptive stimulus processing to both exteroceptive stimuli and the brain’s intrinsic activity. This is possible only if the differences between intrinsic activity and intero- and exteroceptive stimuli is encoded into neural activity. Such relational coding makes possible the assignment of subjective and affective features to the otherwise objective and non-affective stimulus. I therefore consider emotions to be intrinsically affective and subjective as it is manifest in emotional feelings. The relational approach thus goes together with what may be described as neuro-phenomenal approach. Such neuro-phenomenal approach does not only inform emotions and emotional feeling but is also highly relevant to better understand the neuronal mechanisms underlying consciousness in general.

## Introduction

The well-known James–Lange theory determined feelings as perceptions of physiological body changes in the autonomic, hormonal, and motor systems. Once we become aware of physiological bodily changes induced by danger, we feel fear and subjectively experience emotional feelings. James (1884, p. 190) consequently considered bodily changes as central to emotional feelings; “we feel sorry because we cry, angry because we strike, afraid because we tremble, and not that we cry, strike, or tremble, because we are sorry, angry, or fearful, as the case may be.” Modern empirical versions of this theory resurface in current neuroscientific models of emotion as, for instance, in Damasio and others (Damasio, 1999, 2010; Craig, 2003, 2004, 2005, 2009, 2011; Bechara, 2004; Niedenthal, 2007).

Conceptually, the embodied approach to emotion emphasizes the crucial role of the body in emotional feeling. If the body and its vegetative and sensorimotor function play a crucial role in constituting emotional feelings, the body can no longer be considered in a merely objective way but rather as subjective and experienced – the mere Koerper as objective body must be distinguished from the lived body as subjectively experienced body in emotional feeling (Colombetti and Thompson, 2005, 2007; Colombetti, 2008)^1^.

The emphasis on the body raises the question for the role of the environment in constituting emotional feelings. The body stands in direct contact with the environment via its sensorimotor functions which are emphasized in recent body-based, e.g., embodied concepts of emotional feelings (see Niedenthal et al., 2005; Niedenthal, 2007). The body is supposed to represent the environment in sensorimotor terms and it is these bodily representations that are considered crucial in constituting emotional feelings. The environment may have then an indirect and modulatory role via the body in the constitution of the emotional feelings.

One could also imagine that the environment has a direct and constitutive role in emotional feeling; the environment may then directly constitute emotional feeling independent of the body’s sensorimotor (and vegetative) functions. In this case, emotional feelings should be constituted directly by the respective person’s and its brain’s relation to the social environment (see below for definition) rather than indirectly via bodily representations. Since the person-environment relation is crucial here, I call such approach the relational concept of emotional feeling (see Northoff, 2004 for a general outline of such relational approach and Ben-Ze’ev, 1993 for the characterization of perception as relational).

The general aim of the present paper is to review recent human imaging data on emotional feelings in relation to both interoceptive processing and the brain’s intrinsic activity. This will be accompanied by discussing the empirical and conceptual implications of these data which I assume to favor a relational approach to emotions. Such relational concept characterizes emotions and emotional feeling to be intrinsically affective and subjective. Neuronally I assume this to be related to the interaction of the stimuli with the brain’s intrinsic activity, i.e., rest-stimulus interaction (see below for definition). Finally, the empirical and conceptual implications of such relational approach to emotions for consciousness are pointed out.

## Empirical Data: Interoception and Emotional Feeling

### Brain imaging of interoceptive awareness

Recent imaging studies using fMRI investigated neural activity during interoceptive stimulus processing like evocation of blood pressure changes during isometric and mental tasks, heart beat changes and perception, anticipatory skin conductance during gambling, and heart rate modulation during presentation of emotional faces (Critchley, 2005 for a review, Craig, 2002, 2003, 2004, 2009, 2011; Pollatos et al., 2007a,b). These studies observed neural activity changes in the right insula, the anterior cingulate cortex extending from supragenual to dorsal regions (SACC/DACC), and the amygdala. This led to the assumption that specifically the right insula and the SACC/DACC integrally represent autonomic and visceral responses that are transferred from the spinal cord through the midbrain, the hypothalamus, and the thalamocortical pathway to the right insular cortex (Craig, 2002, 2003, 2004, 2009; Critchley, 2005). Based on these results, these regions are assumed to be involved in re-presenting the autonomic and visceral state of the body and thus interoceptive processing. Craig (2002, 2003, 2004, 2009, 2010, 2011) assumes specifically the right insula to be crucially involved which receives autonomic and visceral afferences from lower centers (see above) and re-represents the interoceptive body state in an integrated way. This allows the insula to give rise of a “mental image of one’s physical state” which, according to Craig, provides the basis for subjective awareness of emotional feeling and one’s self as “material me.”

**Figure A1:**
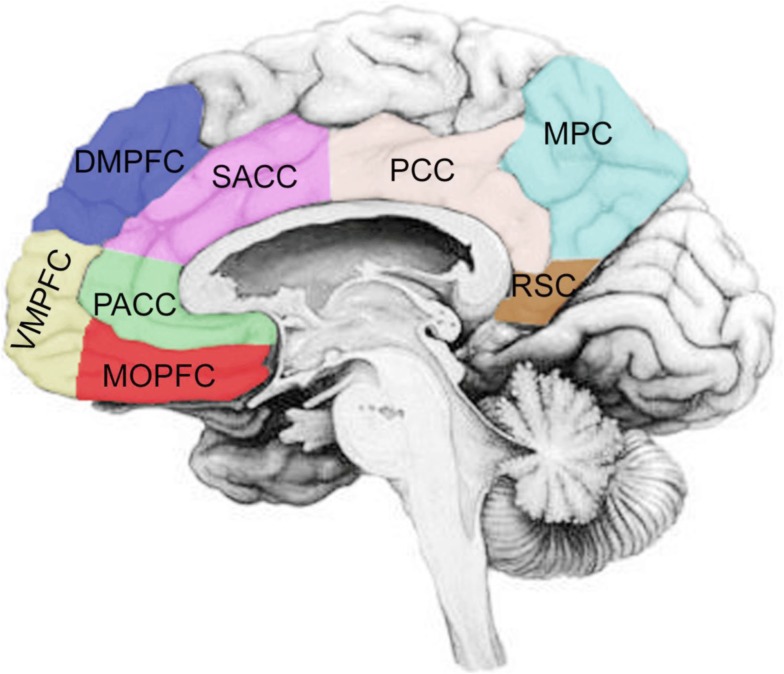
**The figure illustrates schematically the relevant midline regions in the cortex**. The image is a sagittal slice of the brain take in its midline. MOPFC, medial orbitofrontal cortex; VMPFC, ventromedial prefrontal cortex; DMPFC, dorsomedial prefrontal cortex; PACC, perigenual anterior cingulate cortex; SACC, supragenual anterior cingulate cortex; PCC, posterior cingulate cortex; RSC, retrosplenial cortex; MPC, medial parietal cortex.

See Figure A1 in Appendix for the different regions, and Glossarium for the terms.

If these regions mediate interoceptive processing, the question for their role in the subjective experience of bodily and thus interoceptive changes as the basis for emotional feeling arises. Critchley et al. (2004) led subjects evaluate whether the own heart beat was synchronous or asynchronous with an auditory feedback note which allowed to compare interoceptive- and exteroceptive-directed attention. Interoceptive attention to the own heartbeat increased activity in the right insula (and the SACC/DACC and the somatomotor cortex) while exteroceptive attention to the tone suppressed activity in the very same region. Activity in the right insula also correlated with both the performance in the heartbeat detection task and subjective anxiety symptoms which also correlated with each other. These findings suggest close relationship between interoceptive awareness and emotional feeling.

Other studies demonstrated the modulation of these interoceptive stimulus changes by exteroceptive stimuli. Using fMRI, Critchley (2005), for instance, investigated regional neural activity changes during presentation of happy, sad, angry, and disgusted faces. They observed heart rate changes to be dependent upon the emotional category with sad and angry faces inducing the strongest heart rate changes. Emotional face-responsive regions like the right (and left) insula, the SACC/DACC, the midbrain/brain stem, and the right amygdala were also found to be correlating with the changes in heart rate magnitude. These results indicate that different emotions may be mediated by differential interoceptive response patterns which may be mediated by neural activity in the right insula, the SACC/DACC, the midbrain/brain stem, and the amygdala. According to the authors themselves, these results provide support for the hypothesis that interoceptive stimulus processing may be involved in differentiating between different types of emotional feelings.

The group around Pollatos conducted a series of studies on heartbeat perception and emotional feeling. Pollatos et al. (2007a) investigated attention toward heartbeats and cardiovascular arousal; regions implicated in both conditions included the right insula, the somatomotor cortex, the SACC/DACC, and the dorsomedial prefrontal cortex (DMPFC). They observed activity in the right insula and the DACC to be correlating with the degree of interoceptive awareness while negative feelings correlated with the BOLD response of the interoceptive awareness condition in the DACC and DMPFC. Using EEG, they distinguished between good and poor heartbeat perceivers. Good heartbeat perceivers (Pollatos et al., 2005, 2007a,b) showed higher arousal ratings as well as higher P300 amplitudes and slow-wave latency ranges than poor heartbeat perceivers during presentation of emotional pictures.

Taken together, these studies show behaviorally a close relationship between interoceptive awareness, arousal, and emotional feeling. While neuroanatomically, they confirm the involvement of the right insula, the SACC/DACC, and the DMPFC in mediating the relationship between interoceptive awareness and emotional feeling.

### Interoceptive and exteroceptive awareness

The question is whether the above described data support an embodied concept of emotional feeling with exteroceptive stimuli being merely modulatory and instrumental or epiphenomenal. Or whether the data might be interpreted rather in favor of a relational concept of feelings with interoceptive stimuli in relation to exteroceptive stimuli being constitutive and thus central. Presupposing the James–Lange theory, most of the above cited authors have interpreted their data in favor of the interoceptive-based concept. However, I will argue that there are strong arguments which make the data rather compatible with what I call the intero-exteroceptive relational concept of emotional feeling. I argue that there seems to be a mismatch between empirical data and their interpretation in current imaging studies on emotional feelings and interoceptive processing which I want to support by making the three following points.

First, all paradigms employed did not investigate interoceptive stimuli in isolation from exteroceptive stimuli but rather in relation to them. Critchley et al. (2004), for instance, investigated heart beat perception in relation to auditory tones as exteroceptive stimuli while Pollatos et al. (2005, 2007a,b) directly compared both conditions with each other. Neural activity changes assumed to be specific for interoceptive awareness thus reflect a relation or dynamic balance between intero- and exteroceptive processing rather than mirroring isolated interoceptive stimulus processing remaining (more or less) independent of exteroceptive stimulus processing. Dynamic modulation of the right insula activity as observed by Critchley may thus reflect a dynamic balance between intero- and exteroceptive attention in the heartbeat-auditory tone detection task rather than pure interoceptive heartbeat stimulus processing. Such intero-exteroceptive relational concept would thus assume that the above mentioned regions like the right insula, the SACC/DACC, and the DMPFC are rather responsive to changes in intero-exteroceptive balance than to isolated interoceptive changes remaining independent of exteroceptive changes.

Second, neither of the above mentioned studies addressed the question of emotional valence that indicates whether a feeling is positive or negative (see also Colombetti, 2005 for a discussion of the concept of emotional valence). Pollatos et al. (2005, 2007b) did not observe any significant difference between good and poor heartbeat perceivers in terms of their emotional valence ratings while both groups did differ in emotional arousal. Interoceptive awareness may thus be linked to emotional arousal and subjective experience of emotional intensity while it apparently does not seem to determine the valence of the emotional feeling. Regions that have been associated with emotional valence, as distinguished from emotional arousal, include the medial orbitofrontal cortex (MOFC), the subgenual and pregenual anterior cingulate cortex (PACC), and the ventromedial prefrontal cortex (VMPFC; Craig, 2002, 2009; Phan et al., 2002; Critchley, 2005; Kringelbach, 2005; Grimm et al., 2006).

Interestingly, these regions are densely and reciprocally connected with the right insula, the SACC/DACC, and the DMPFC that are supposed to represent the body’s interoceptive state (Ongur and Price, 2000). The connectivity pattern thus argues strongly in favor of the intero-exteroceptive relational concept of emotional feeling which seems to make isolated interoceptive processing and thus an interoceptive-based concept of emotional feeling rather unlikely. What however is needed to further support this point are investigations of both regional activity and connectivity patterns during intero- and exteroceptive stimulus processing (see Hurliman et al., 2005 for some first support).

Third, Pollatos et al. (2005, 2007b) investigated the temporal course with EEG during heartbeat perception task. They observed that good heartbeat perceivers showed higher heart-evoked potentials and stronger dipole strength in cortical sources that included the SACC/DACC, the right insula, the DMPFC, and the secondary somatosenory cortex when compared to poor heartbeat perceivers. Interestingly, they also observed the dipole sources in the SACC/DACC and DMPFC to occur earlier (around 280 ms) than the ones in the insula and the somatosensory cortex (around 370 ms). A similar temporal distribution is suggested by Tsuchiya and Adolphs (2007) who assume involvement of subcortical regions like brain stem nuclei and hypothalamus that mediate interoceptive stimuli to occur after and later than activation in higher regions like the DMPFC. If the interoceptive-based model were true, one would rather expect the opposite temporal pattern with early insula and somatosensory involvement and late SACC/DACC and DMPFC involvement.

Late SACC/DACC and DMPFC involvement may then reflect some abstract internal cognitive evaluation of interoceptive stimulus processing with consecutive top-down modulation of interoceptive brain regions as interpreted by advocates of the interoceptive-based concept (Craig, 2002, 2009; Tsuchiya and Adolphs, 2007). What is the role of the SACC/DACC and the DMPFC? These higher cortical regions have been associated with processing of higher-order exteroceptive stimuli particularly those that are highly self-related to the organism (Northoff and Bermpohl, 2004; Northoff et al., 2006).

The fact that these regions are apparently implicated from early on in interoceptive awareness gives some though indirect support to the assumption that exteroceptive stimuli are involved early in interoceptive processing. Such early involvement indicates that the role of exteroceptive stimulus processing goes beyond mere modulation of interoceptive processing which would be better compatible with late involvement. In other terms, early involvement of these regions may indicate that interoceptive stimulus processing is coded in relation to exteroceptive stimuli going beyond mere modulation of the former by the latter. The observed early spatio-temporal pattern may thus reflect neural coding of the relationship between intero- and exteroceptive stimulus processing, i.e., their actual balance. Otherwise there would be no need for regions predominantly associated with exteroceptive stimulus processing to be implicated so early. While it seems to be less compatible with the assumption of primarily independent interoceptive processing that becomes secondarily modulated by exteroceptive stimuli.

Finally, direct empirical support for intero-exteroceptive convergence comes from a recent study by Farb et al. (2012). He investigated interoceptive awareness (i.e., attention to breathing rate) and exteroceptive awareness (i.e., visual attention) in the same subjects. While both intero- and exteroceptive awareness yielded dissociable networks (i.e., visual cortex and posterior insula), they overlapped in especially the anterior insula. Unlike the posterior insula that responded strongly to interoceptive awareness, the anterior insula activity was as much predicted by exteroceptive awareness as interoceptive awareness. Hence, there seems to be intero-exteroceptive convergence in especially the anterior insula with both being integrated in the middle insula as bridge from posterior to anterior parts of the insula.

## Empirical Implications: Connectivity and Coding

### Anatomical connectivity and intero-exteroceptive convergence

The MOFC and the VMPFC have been demonstrated to be implicated in interoceptive processing. Using biofeedback arousal and relaxation tasks in fMRI, Nagai et al. (2004) demonstrated that resting state activity in the VMPFC and MOFC co-varied with the basal level of sympathetic skin conductance. While regions like the SACC/DACC, the insula, and the hypothalamus were related to the rate of change in skin conductance. The level of neural activity in VMPFC and MOFC, which are part of the so-called anterior cortical midline structures (aCMS), may thus represent the basal sympathetic or autonomic tone independent of some actual stimuli. Since the aCMS have been shown to be modulated also by exteroceptive stimuli, neural activity within these regions may mirror a dynamic balance between attention to extero- and interoceptive stimuli (see also Nagai et al., 2004). This assumption is well compatible with the connectivity pattern of these regions.

The MOFC and VMPFC as the entrance door to the aCMS receive connections from all regions associated with primary and/or secondary exteroceptive sensory modalities (olfactory, gustatory, somatosensory, auditory, and visual; see Rolls et al., 1999; Barbas, 2000; Rolls, 2000; Damasio, 2003, 2010; Kringelbach and Rolls, 2004). The aCMS are also densely connected to regions (insula, hypothalamus, and nuclei in the brain stem as such PAG, colliculi, etc.) processing interoceptive sensory signals; these include the proprioceptive and vestibular senses, the visceral sense, and the sense of the interoceptive milieu which can be taken together with that of pain and temperature (Carmichael and Price, 1996; Price, 1999; Rolls et al., 1999; Rolls, 2000; Damasio, 2003, 2010; Barbas, 2004; Kringelbach and Rolls, 2004). The aCMS, especially the MOFC, VMPFC and SACC/DACC, are also connected to regions associated with distinct functional domains including motor (premotor and motor cortex, basal ganglia), cognitive (lateral prefrontal cortex), and emotional (amygdala, brain stem) domains (Carmichael and Price, 1996; Rolls et al., 1999; Barbas, 2000; Ongur and Price, 2000; Rolls, 2000; Kringelbach and Rolls, 2004). Due to such extensive intero- and exteroceptive connections, the MOFC and VMPFC (and, in conjunction with the amygdala) can be characterized as polymodal convergence zone (Rolls et al., 1999; Rolls, 2000; LeDoux, 2002; Schore, 2003).

This connectivity pattern predisposes the aCMS for neural processing irrespective of the sensory modality of the respective stimulus, i.e., supramodal processing. The assumption of supramodal processing in aCMS is supported by results from imaging studies. Emotions in either exteroceptive modality (visual, auditory, gustatory, olfactory) induce neural activity in various regions of the aCMS (see above as well as Phan et al., 2002; Northoff and Bermpohl, 2004). Moreover, processing of interoceptive stimuli induces also activation in aCMS regions like MOFC, VMPFC, and ACC (Craig, 2002, 2003, 2004, 2009; Wicker et al., 2003; Critchley et al., 2004; Nagai et al., 2004). Finally, stimuli from different origins, i.e., of different sensory modalities or of different functional domains (motor, emotional, cognitive, and sensory) induced analogous activation in aCMS (Northoff and Bermpohl, 2004; Northoff et al., 2006).

Taken together, both connectivity pattern and imaging data suggest that neural processing in aCMS is supramodal and domain-independent: what apparently matters for inducing neural activity in the aCMS is not so much the modality or domain, i.e., the origin of the stimulus, as either intero- or exteroceptive or cognitive, motor, sensory, or emotional. Instead it is important how the neural activity in the aCMS is related to the respective intero- or exteroceptive stimulus (see below for further discussion).

In addition to the aCMS, subcortical midline regions like the periaquaeductal gray (PAG), the colliculi, the dorsomedial thalamus, and the ventral striatum may also be considered in processing interoceptive stimuli in relation to exteroceptive ones. Panksepp (1998; and also Damasio, 1999, 2010), for instance, assumes that these regions are crucial in constituting emotional feelings. Since the very same regions are also characterized by strong motor connections both afferent and efferent, he and others like Ellis (2005; unlike Damasio who assumes a sensory-based view of feelings) assume emotional feeling to be motor-based. This is well compatible with Panksepp’s characterization of emotional feeling as reaching-out to the environment thus reflecting what I called the relational concept of emotional feeling.

Unfortunately, subcortical regions have often been neglected in imaging studies of emotions which, at least in part, may be due to the fact that neural activity in these regions is rather difficult to reliably visualize in current imaging techniques like fMRI. However, animal experiments demonstrate the crucial role of these subcortical midline regions in constituting emotional feelings (Panksepp, 1998, 2005). Future studies in humans are thus needed to investigate subcortical neural activity during emotional feeling in order to bridge the current gap between animals and humans. Furthermore, the relationship between emotional feeling and motor function also needs to be investigated in detail by, for instance, investigating emotional feeling in dependence on variation of motor function and its neural underpinnings (and vice versa).

### Translational versus relational coding

What is the implicit presupposition that drives most of the above cited authors to interpret their data in favor of the James–Lange theory? They seem to presuppose a clear-cut distinction between intero- and exteroceptive stimulus processing with both systems being separate, distinct, and only interacting at specific node points. According to such view, exteroceptive stimuli are translated into interoceptive stimulus processing whose perception, in turn, is supposed to induce feeling. Exteroceptive stimuli thus have at best an only indirect and mediated impact on emotional feeling in that they must first be translated into interoceptive stimulus processing before they can modulate feelings. I therefore call this model the interoceptive-based translational concept of feeling. Since exteroceptive stimuli have only an indirect and mediated, the interoceptive-based translational concept attributes no constitutive role of exteroceptive stimuli and the environment thus presupposing an “embodied” concept of emotional feeling.

**Figure 1:**
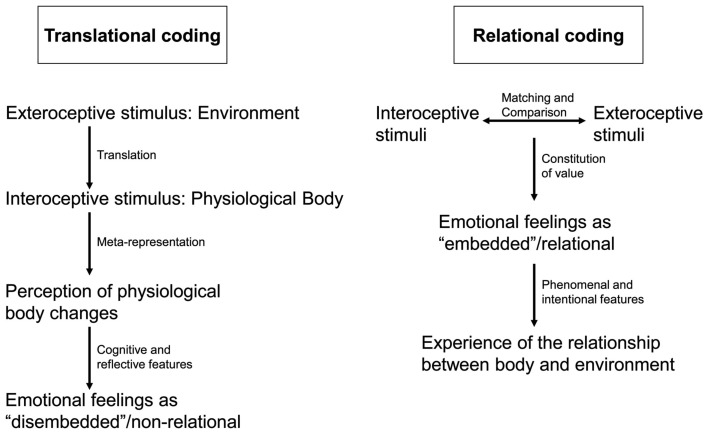
**The figure compares two different ways of neural coding in emotional feeling**. On the left side translational coding describes how intero- and exteroceptive stimuli are separately represented and meta-represented in the neural activity of the brain. This meta-representation is then perceived which following Damasio and the James–Lange theory leads to emotional feelings. This is different in relational coding on the right side. Here intero- and exteroceptive stimuli are coded in relation to each other with this relation resulting in emotional feeling and subsequent experience of the relationship between body and environment.

However, anatomical connectivity suggests otherwise. Throughout the brain at all levels both subcortical and cortical and especially in the subcortical-cortical midline system there is convergence between intero- and exteroceptive inputs. This is especially true for regions like the colliculi, the PAG, the tectum, and the aCMS where both intero- and exteroceptive afferences converge onto common neurons (see Panksepp, 1998, 2005; Rolls et al., 1999). This suggests that interoceptive stimuli are not only modulated by exteroceptive stimuli at specific node points but rather that the relation, e.g., the degree of convergence and divergence, between intero- and exteroceptive stimuli is coded in neural activity in the subcortical-cortical midline regions. Exteroceptive stimuli are not translated into interoceptive stimulus processing but rather directly and unmediated related to them and it is this relation that seems to be coded in neural activity. I therefore call this model the intero-exteroceptive-based relational concept of feelings (see also Figure 1).

Is there any empirical evidence in favor of the intero-exteroceptive relational model of neural coding? Critchley (2005, p. 162), one of the main investigators of interoceptive processing in imaging, states, that the “right insula maps bodily arousal states” and “it does so contextually” which therefore “represents an integration of external emotional information with peripheral states of arousal” (Critchley, 2005, p. 759). What seems to be coded in the brain is not so much the interoceptive stimulus itself but its relation to the respective exteroceptive stimulus. If neural activity codes the actual relationship and balance between intero- and exteroceptive stimuli, one would expect strong contextual dependence of emotional feelings.

The constitution of the emotional feeling, the type of feeling, should then depend on the respective emotional context which implies that different contexts may lead to different types of emotional feelings even in identical situations. In other terms, the environmental context does not only modulate emotional feelings but actively participates in constituting emotional feelings. This is well in accordance with the Schacter/Singer experiments where different contexts resulted in different types of emotional feelings. If the role of the context were merely modulatory, subjects would not have shown completely different and opposing emotional feelings in the two situations but rather variants of the same feeling. These experiments thus lend further support to the assumption of a constitutive role of the environmental context in emotional feelings (rather than remaining merely modulatory).

How are intero- and exteroceptive stimuli related and balanced with each other in relational coding? Rather than coding the intero- or exteroceptive stimulus itself, the degree of correspondence between intero- and exteroceptive stimuli is coded. If, for instance a lion approaches, the heart rate may increase, which may signal strong correspondence and convergence between intero- and exteroceptive stimuli. This consecutively leads to the constitution of a corresponding emotional feeling, the feeling of fright and anxiety. If, in contrast, the approach of the lion is not accompanied by heart rate increases, as for instance if one is not clear whether the lion is real or not, there may be a mismatch between intero- and exteroceptive stimuli. This may result in a different emotional feeling, the feeling of doubt and hesitation. The degree of convergence and divergence between intero- and exteroceptive stimuli may thus determine the kind of emotional feeling. That is well in accordance with the relational concept rather than with the translational one that claims for an interoceptive- and thus bodily based approach.

Taken together, I assume that our brain’s design is such that there is no way for interoceptive stimuli other than to be processed in relation to exteroceptive stimuli and vice versa. Interoceptive stimulus processing remaining isolated, unrelated and independent from exteroceptive stimulus processing is consequently assumed to remain (principally) impossible. This implies what I call intero-exteroceptive relational coding while it excludes interoceptive-based translational coding. What does this imply in experimental regard? The experimental efforts to isolate interoceptive stimulus processing and to search for its specific neural correlates may be futile since exteroceptive stimulus processing may always already be implicated in interoceptive stimulus processing. One may better focus on experimentally investigating different intero-exteroceptive stimulus configurations and thus different constellations between body and environment as nicely demonstrated in the Schacter/Singer experiments (see Northoff, 2012a for more details on the question of neural coding).

## Conceptual Implications: Relational Approach to Emotional Feeling

### Relational concept of emotional feeling

The philosopher Hurley (1998, pp. 10, 341–342, 362–364) distinguishes between instrumental and non-instrumental dependence (see also Colombetti, 2008 who also applies this distinction) with regard to the relationship between input and output in perceptual content. If the relationship between input and output is indirect and thus merely instrumental, changes in perceptual content are dependent upon changes in the input. Every change in motor output has to modulate sensory input in order to have an impact on perceptual content implying that the output can not change independently of the input: “This kind of dependence of perceptual content on output is merely instrumental. It operates via changes in input; changes in output are a means to changes in input” (Hurley, 1998, p. 10).

What does this mean with regard to emotional feelings and their relation to the environment? Presupposing instrumental dependence, the environment can impact emotional feelings only indirectly via the body, i.e., by being represented either in the body’s sensorimotor (and vegetative) functions or in those brain regions that register the body’s sensorimotor (and vegetative) functions. The latter approach is, for instance, advocated by the proponents of Damasio’s theory of emotional feeling where the relation between body and environment remains at best modulatory (and contributing but not as constitutive). This is nicely reflected in a quote from a recent paper about emotion and consciousness: “Here, we follow the common view that emotion and consciousness emerge as a result of neuronal activity in the brain, but some accounts view emotions or consciousness as relationships between an organism and its environment (here we acknowledge such relationships as contributing but not as constitutive)” (Tsuchiya and Adolphs, 2007, p. 159; see also Bechara and Naqvi, 2004).

Non-instrumental dependence, in contrast, is described by Hurley as direct dependence of perceptual content on motor output independent of sensory input; even if the sensory input remains the same and fixed, perceptual content can vary depending on motor output. This means that motor output has direct access to perceptual content independent of sensory input and therefore no longer operates indirectly via sensory input as in instrumental dependence; instead, perceptual content may vary in orientation on motor output independent of sensory input and thus directly.

What does such non-instrumental or constitutional, as I will call it in the following (see also Colombetti, 2008), dependence imply for the relationship between body and environment in emotional feeling? If the relationship between emotional feeling and environment is direct and therefore constitutional, i.e., non-instrumental, changes in the environment should be able to impact and constitute emotional feelings independently of the body’s sensorimotor representation. The environment itself may then directly involved in constituting emotional feelings. Thereby, the concept of environment is meant here in a social sense, social environment, as distinguished from the merely physical world (or physical environment).

This has empirically been paradigmatically exemplified in a recent study on reward (Fliessbach et al., 2007). Two subjects a and b were simultaneously scanned while receiving rewards. While the reward for the subject a was fixed, the one for subject b was varied; this and the converse case, increasing rewards for subject a and fixed rewards for subject b, allowed to investigate its impact of the environment, i.e., subject b, on subject a. Interestingly, emotional feelings and neural activity in reward circuitry in subject a did not so much depend on the size of the reward it received but rather on the relation of or balance to its own reward when compared to the one received by subject b. If, for instance, subject a received 60$ and subject b only 30$, subject a showed happiness and increased reward circuitry activation. If, in contrast, subject b received 120$ with subject a still receiving 60$, subject a no longer showed happiness and increased neural activity in reward circuitry. Though sensorimotor input was exactly the same for subject a in both cases (only subject’s b reward amount changed), playing the same game and receiving the same reward, emotional feelings, and neural activity in reward circuitry differed in dependence on the amount of reward subject b received when compared to the amount subject a received.

This means that, to put it into conceptual terms, emotional feelings and neural activity in subject a were not merely instrumentally dependent upon the social environment (since then changes in subject b could have impact subject a only if they had changed subject’s a reward) but rather instrumentally or constitutionally. More specifically, it is the relationship between person and (social) environment, the actual difference or balance between subjects’ a and b rewards, that seemed to determine emotional feelings and neural activity. It is such constitutional, i.e., non-instrumental, dependence of emotional feelings on the social environment and its relationship to the person that I will characterize as the relational concept of emotional feelings^2^. Such intrinsic linkage between emotional feelings and the social environment is empirically further supported by the observed overlap between emotion processing and social processing (like social intentions; see Ciaramidaro et al., 2007) in especially aCMS like the anterior cingulate cortex and the DMPFC (see Schilbach et al., 2012). These (and other) data end strong support to an intrinsically social and thus relational concept of emotional feeling.

### Embedded approach to emotional feeling

The relational approach shifts the focus of attention from the body, as in the embodied approach, to the role of the environment in emotional feelings. Rather than modulating emotional feelings indirectly via bodily representations, the environment is supposed to be involved directly in constituting emotional feelings. How does the person-environment relation account for the variety of different specific emotional feelings? The lack of specificity concerning distinct emotions has often been criticized in feeling theories like the James–Lange theory (see also Niedenthal et al., 2005). Autonomic bodily changes like arousal are rather unspecific reactions that do not allow to distinguish between distinct emotions. This criticism has been furnished by the Schachter and Singer (1962) experiments demonstrating that subjects with autonomous nervous system stimulation, as induced by epinephrine, experienced the resulting arousal as either anger or euphoria in dependence on the respective context (they were placed in a room with either an angry or happy actor).

The conclusion is often drawn that physiological bodily changes and arousal themselves remain unspecific and cannot contribute to determine specific emotions); determination and distinction of specific feelings can consequently not be based upon physiological bodily changes but must be found elsewhere. This argument of the lack of specificity of bodily representations has been countered in different ways by referring to motor, cognitive, or neural representation. Zajonc (1998, 2000), for instance, claims that the motor system allows for extremely subtle distinctions which means that even a number of limited bodily states can support a very large number of representational distinctions of distinct emotional feelings. Rather than referring to motor capacities, cognitive theories, e.g., appraisal theories (Solomon, 2004; and also Schachter and Singer, 1962) resort to cognitive representations and higher-order cortical brain functions which may allow for a much more fine-grained distinction between different emotional feelings.

Damasio (1999, 2003, 2010) suggested a middle way between motor and cognitive representation. He focuses on those subcortical brain regions that register physiological bodily states which may allow for a wider representational spectrum than the muscles and viscera themselves that are actually represented in the respective neural states. All these approaches have in common that they still presuppose representation of emotional feelings in motor, cognitive, or neural-subcortical functions.

The relational approach, in contrast, claims that the wide variety of different emotional feelings may ultimately be traced back to the relation between person and environment rather than to motor, cognitive, or neural-subcortical representation. Since an abundant variety of different person-environment relations are possible, different emotional feelings can be constituted. The question for the specificity of emotional feelings is thus traced back to the possible (and impossible) person-environment relations rather than to the representational capacities of specific functions, i.e., motor, cognitive, or physiological-registering.

If emotional feelings are intrinsically relational, i.e., depending upon the person-environment relation rather than some representational capacities in motor, cognitive, or neural-subcortical function, one would expect different feelings to reflect different kinds of person-environment relationships. Ratcliffe (2005, 2008) does indeed assume exactly this and assumes what, relying on Heidegger’s phenomenology, he calls “existential feelings.” “Existential feelings” include feelings of homeliness, belonging, separation, unfamiliarity, power, control, being part of something, being at one with nature, and “being there.” These feelings have in common that they describe “ways of finding ourselves in the world” which metaphorically circumscribes what I called the person-environment relation. What Ratcliffe calls existential feeling presupposes what I here advance as relational concept of emotional feeling. How does the person and thus the subject come into play in emotional feelings? This will be the focus in the next sections.

## Empirical Data: Intrinsic Activity and Emotional Feeling

### Emotions and resting state

I so far focused on the relationship between intero- and exteroceptive stimulus processing in emotional feeling. Neurobiologically this was supposed to be related to the anatomical convergence between the respective pathways and the kind of coding, i.e., relational coding rather than translational coding. While conceptually this implied a shift from the embodied concept of emotional feeling to a relational concept (or as the philosophers may want to say an extended concept). This pointed out the intrinsic relation to emotional feelings to the environment as bridge between body and environment.

What remains unclear though are two aspects: why are emotional feelings not objective but rather subjective as manifest in an emotional experience? And why are emotional feelings affective and thus emotional? Taking a purely logical stance one could well imagine mere feelings without any emotions. Both questions dent deeply in various domains of research including consciousness (see Northoff, 2012a,b) which though I will avoid here to keep matters simple. I will here focus only on some neurobiological mechanisms while leaving the philosophical implications open. In order to shed some light on these questions, I turn to recent results about the relation between resting state activity and emotions.

A recent study by Sreenivas et al. (2012) investigated different emotional faces (sad, happy, neutral) in fMRI and focused thereby predominantly on the midline regions of the default-mode network (DMN). They demonstrated that sad faces induced a higher degree of deactivation, i.e., negative signal changes in the VMPFC, the PCC, and the precuneus when compared to happy faces. In contrast, activation and thus positive emotional signal changes were observed in the lateral fronto-parietal regions (except in left middle frontal gyrus). Finally, functional connectivity pattern also differed between sad and happy emotions for the connections between the midline and the lateral regions with VMPFC, PCC, and precuneus being central nodes.

While this study demonstrates that emotions are associated with midline regions that show high resting state activity, it leaves open whether that is related to intero- or exteroceptive stimuli. This was tested for by a study by Wiebking et al. (2011) from our group. Subjects had to perform the above mentioned intero-exteroceptive awareness with long resting state intervals (8–13 s) in-between. These served to subtract both intero- and exteroceptive signal changes from the resting state which, as expected, yielded higher activity changes in the midline regions. We then determined the degree of deactivation during both intero- and exteroceptive awareness. That yielded stronger deactivation in exteroceptive awareness when compared to interoceptive awareness.

How is all that related to emotions? For that Wiebking et al. (2011) included psychological measures of emotions (i.e., the Florida Affect battery and the Beck Hopelessness scale) and correlated them with the signal changes in the midline regions during the different conditions. Interestingly, we did not observe any correlation of the emotion measures with the midline signal changes during interoceptive awareness alone. Instead, the emotion measures significantly correlated with especially signal changes in VMPFC, DMPFC, and PCC during rest and exteroceptive awareness: The stronger the emotion score, the smaller the degree of deactivation in the midline regions thus being closer to the resting state activity level. In contrast, no correlation was observed with signal changes during interoceptive awareness. Hence, these results underline the central importance of intrinsic and thus resting state activity for emotions.

### Modulation of resting state by emotions

These results show the strong association between resting state activity and emotions. They though leave open whether emotions can also modulate resting state activity or whether the latter predict the former. Several recent studies demonstrated the prediction of stimulus-induced activity by the preceding resting state activity implying rest-stimulus interaction (see Northoff et al., 2010 for a review). This was mainly shown in the in the sensory domain while, as to my knowledge, such studies are not yet available in the domain of emotions. There are though a couple of studies that show the reverse, modulation of resting state activity by preceding emotions.

Focusing on emotions, Eryilmaz et al. (2011) investigated the impact of fearful, joyful, and neutral movie clips (50 s presentation) on subsequent resting state activity (90 s eyes closed). They asked the participants after the resting state period about their thoughts. This revealed that the subjects’ personal relevant issues in their thoughts were increased after neutral movies, less increased after joyful movies, and significantly decreased after fearful movies. These results show a clear behavioral or better psychological effect of emotions on the thought contents in subsequent resting state periods; fearful movies seem to leave apparently the strongest traces in the subsequent resting state’s thought contents.

Neuronally, they showed that the resting state periods after fearful faces showed higher neuronal activity in subcortical regions (pallidum, anterior thalamus, hypothalamus) than the ones following neutral movies (rest after fearful larger than rest after neutral). Most interestingly, the reverse comparison (rest after neutral larger rest after fearful) revealed higher signal changes in various regions of the DMN (VMPFC, PACC, DMPFC, STG). This means that the inclusion of fearful emotions in the preceding movie had a clear effect on the level of subsequent resting state activity. The stronger resting state effects of the preceding emotional movies are further confirmed by the more delayed recovery of the signal changes during the resting state period (90 s) after emotional movies.

This study clearly demonstrates that emotions have an impact on the subsequent resting state thus implying what we coined as stimulus-rest interaction (see Northoff et al., 2010). This was also observed in another study. Veer et al. (2011) investigated a psychological stress task in healthy subjects and scanned them in their resting state one hour later in fMRI. This revealed increased functional connectivity from the amygdala to the cortical midline structures like the MPFC, the PCC, and the precuneus. This indicates that psychological stress implicating strong and negative emotions can affect the subsequent resting state activity thus implying stimulus-rest interaction.

Taken together, these studies demonstrate the close relationship between resting state activity and emotion-related activity. This seems to be especially apparent in the midline regions as core nucleus of the DMN. The high intrinsic activity in these regions seems to be closely related to emotion processing in though yet unclear ways. Different emotions seem to modulate the degree of stimulus-related deviation from the high resting state activity in different ways. The close relationship between emotions and resting state is further supported albeit indirectly by the observation of severe resting state alterations in major depressive disorder (see Alcaro et al., 2010; Northoff et al., 2011; for recent overviews).

## Empirical Implications: Intrinsic Activity and the Subjective Nature of Emotional Feeling

In order to better understand the potential role of the brain’s intrinsic activity in emotional feeling, we need to go back to the psychological level. For that I turn to two of the major proponents of emotional feeling, Jaak Panksepp and Jim Russell, and how they conceptualize especially the subjective-experiential component of emotional feeling. This will be then linked in subsequent sections to the above described findings of the close relationship between intrinsic activity and emotions.

### Panksepp and russell on emotional feeling

Based on the centrality of affect and emotions, Panksepp (1998, 2010) developed a neuroscientifically based theory of primary process affects as raw emotional feelings which he associates with evolutionary ingrained subcortical circuits. Russell (2003) shifted from an earlier Psychological Construction Theory of emotions to the assumption of what he calls “Core Affect” as a basic and foundational unit (or building block) of any specific emotional feeling. While Panksepp’s concept of “primary process affect” overlaps at least conceptually (and also to some degree empirically) with Russell’s concept of Core Affect (see especially Russell’s p. 6–7 commentary on Panksepp), they are not the same.

Panksepp distinguishes between three distinct kinds of primary process affects, homeostatic, sensory, and emotional. Homeostatic affect provides information about the body and thus interoceptive stimuli, sensory affect is related to exteroceptive stimuli, and emotional affect is associated with the brain (or BrainMind as Panksepp says) and hence with what one may call “neural stimuli.” These distinctions make it clear that primary process affect is linked with stimuli generally, and more specifically with stimuli of different origins, be they of bodily (i.e., interoceptive), environmental (i.e., exteroceptive), or neural origin. Hence, primary process affect must be somehow assigned to stimuli since otherwise Panksepp could not associate primary process affect with stimuli of such different origins. I call such association of stimuli with affect or primary process affect “affective assignment” meaning that a stimulus of whatever origin can be assigned affect.

Analogous to Panksepp, Russell must also presuppose affective assignment though in a slightly different way. He does not associate what he describes as Core Affect itself with a specific type of stimulus since unlike Panksepp he does not speak of sensory, homeostatic, or emotional Core Affect. Instead, Core Affect is continuously present independent of the presence or absence of particular stimuli. One though has to mention that Russell seems to refer here only to the absence of exteroceptive stimuli since he does not explicitly talk about interoceptive or even neural stimuli in this context. This means that it cannot be excluded that Core Affect may be related to the assignment of affect to either neural or interoceptive stimuli. Hence Russell’s concept of Core Affect would then also presuppose what I call affective assignment.

Rather than to interoceptive stimuli, Russell explicitly refers to the assignment of affect to exteroceptive stimuli when he describes the transition from Core Affect to emotional episodes and emotional meta-experience. In the moment when the continuously present Core Affect is related to an episodically occurring exteroceptive stimulus, an emotional episode and meta-experience may occur. This however is possible only if the Core Affect is linked and thus assigned to the exteroceptive stimulus thus presupposing what I here call affective assignment.

While both Panksepp and Russell seem to presuppose the assignment of affect to stimuli, the exact functional mechanisms that enable and predispose such affective assignment remain unclear in their accounts. What functional mechanisms are necessary to enable and predispose the transformation of a non-affective stimulus into an affective one? I call this the “non-affective-affective transformation.” The “non-affective-affective transformation” raises the question how it is possible that a stimulus is suddenly associated with either Core Affect or primary process affect. It is especially worthwhile to consider that the stimulus of interoceptive, exteroceptive, or neural origin must be non-affective. Hence “non-affective-affective transformation” raises the question: what kind of functional mechanisms and neural input the brain must provide in order to assign affect to the stimulus.

The question about the “non-affective-affective transformation” raises another issue. Both Panksepp and Russell consider affect to be essentially subjective rather than objective. Panksepp refers to primary process affect as subjective by describing it as an “internal experience” while Russell describes Core Affect as subjective in the sense of a private experience. Hence, Panksepp distinguishes internal from external and Russell private from public when they characterize Core Affect or primary process affect as subjective rather than objective. One should need to make a conceptual remark here. The meaning of the term subjective refers here only to the experience of affect, it does not say anything about the underlying neuronal mechanisms that may well be objective.

This raises the question how affective assignment makes it possible to transform the originally objective stimulus, interoceptive, exteroceptive, or neural, into a subjective one. Hence, “non-affective-affective transformation” is not limited to transforming a non-affective into an affective stimuli but with transforming the objective into a subjective stimulus. I therefore speak of “objective-subjective transformation.” “Objective-subjective transformation” raises the following question: how do the neuronal mechanisms enable the transformation of an objective stimulus into a subjective one? Such that, in conjunction with “non-affective-affective transformation” the stimulus can be subjectively and thus internally and privately experienced.

### “Objective-subjective transformation”

**Figure 2:**
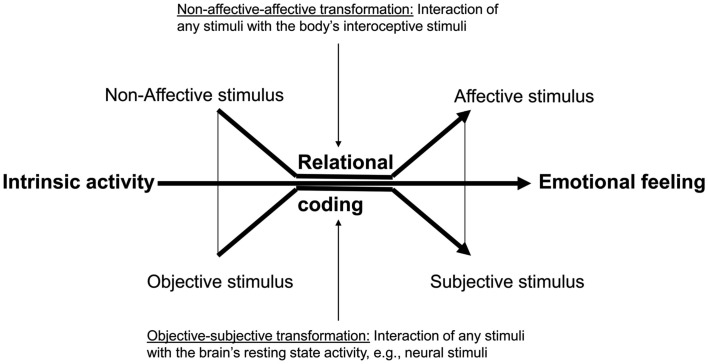
**The figure shows the relationship between intrinsic activity and stimuli (on the left) and emotional feeling (on the right)**. The intrinsic activity of the brain interacts with the stimuli that are by themselves non-affective and objective. That rest-stimulus interaction leads to the assignment of affect and subjectivity to the stimulus resulting in emotional feeling.

Both Russell and Panksepp seem to presuppose some kind of intrinsic stimuli to be crucial in generating affect. Russell does so by explicitly distinguishing Core Affect from extrinsic stimuli and related emotional episodes, while Panksepp argues that neural activity in the subcortical circuits is not dependent upon extrinsic stimuli, i.e., exteroceptive stimuli. This means that both must presuppose some kind of intrinsic activity for the generation of affect (Figure 2).

What could such intrinsic activity be? One may assume it is that activity that can be observed in the absence of any extrinsic stimulation by either intero- or exteroceptive stimuli. Intrinsic means then that the origin of that activity must be traced back to the brain itself as distinguished from body and environment. One may refine such intrinsic activity as the brain’s resting state activity, or that activity in the brain in the absence of any intero- and exteroceptive stimuli (see Northoff et al., 2010). And it is such resting state activity as intrinsic activity that can be observed in all brain regions cortical and subcortical (see Northoff et al., 2010).

The fact that resting state activity is present throughout the whole brain means that there may already be some neural interactions between the different brain regions within the resting state itself. For instance the resting state activity level in the sensory cortex may interact with the resting state activity level in the subcortical regions so that one may want to speak of rest–rest interaction. The above described results lend clear empirical support to the assumption that such resting state activity in especially the midline regions is central for emotions and most likely for emotional feelings.

And there is further interaction. As soon as an inter- or exteroceptive stimulus enters the brain it interacts with the brain’s resting state activity level thus yielding what can be called rest-stimulus interaction (Northoff et al., 2010). Such rest-stimulus interaction may be specified according to the stimulus type either rest-interceptive stimulus interaction or rest-exteroceptive stimulus interaction (which in the following I will describe as rest-intero and rest-extero interaction).

How do the three types of interaction, rest–rest, rest-intero, and rest-extero relate to affective assignment and more specifically to the non-affective-affective transformation and the objective-subjective transformation? The resting state activity level is different not only between different regions but even more importantly, different between different persons. This means that the same stimulus encounters a different brain in different persons meaning it must interaction with a different resting state activity level. Rest-stimulus interaction individualizes the stimulus and adapts its processing according to an individual brain’s resting state activity level. Due to the individual resting state activity level and its impact on rest-stimulus interaction, the stimulus is processed in a very individual and ultimately private way.

By individualizing and privatizing the stimuli with respect to the brain’s actual resting state activity level, the originally objective stimulus is transformed into a subjective one. Hence what I called above objective-subjective transformation may correspond on a functional level to rest-stimulus interaction. Any processing of the stimulus, interoceptive or exteroceptive, in relation to the brain’s resting state activity level (and hence its neural stimuli) may privatize and individualize the stimuli, and thereby transform it from an objective to a subjective one.

Russell and Panksepp may now want to argue that this accounts only for half of the story. Panksepp may say that this leaves emotional affects as based on the stimuli from the BrainMind itself and hence its neural stimuli out; this may be so because rest-stimulus interaction concerns only the interaction with intero- and exteroceptive stimuli. Hence, my assumption of rest-stimulus interaction corresponding to objective-subjective transformation may well account for what Panksepp calls homeostatic affects and sensory affects, but not emotional affects.

Russell may want to make an even stronger point. My assumption of rest-stimulus interaction misses Core Affect altogether because Core Affect is neither related to interoceptive nor exteroceptive stimuli. Instead the core affect precedes both kind of stimuli that becoming relevant only in emotional episodes. Hence, my assumption that rest-stimulus interaction corresponds to objective-subjective transformation may hold for emotional episodes and emotional meta-experience but not for Core Affect itself.

This however is to neglect that the brain’s resting state can interact with itself, as for instance the resting state activity level in the subcortical circuits interacting with cortical regions. There may thus be what could be called “rest–rest interaction” where the neural stimuli of one particular region’s resting state may interact with those of another region. Recent imaging data show that such rest–rest interactions do indeed occur (see Northoff et al., 2010 for recent review). In the case of such rest–rest interaction, the resting state activity level of one network is set against that of another network.

### Subjective character of emotions

This has major implications for the conceptual characterization of emotions. Any stimulus, internal, external, or neural, cannot avoid but to interact with the brain’s resting state activity. If that very same resting state activity individualizes and privatizes stimuli and their encoding into neural activity, any emotions must be individualized and privatized. That though means that any emotions must necessarily be subjective meaning that it cannot avoid objective-subjective transformation. There is consequently no emotion without emotional feeling with the latter being at the very core of the former. This nicely corresponds to what Russell describes as “Core affect” and Panksepp as “primary process affect” and, more generally as “BrainMind.”

To empirically support this assumption, future studies are needed to test whether the preceding level of resting state activity predicts the degree of especially the subjective-experiential component of emotions, i.e., the emotional feeling. I would hypothesize that the preceding resting state activity predicts especially the subjective-experiential component of emotions. While other components like the vegetative and the cognitive aspects of emotions may rather be predicted by the degree of stimulus-induced activity itself.

One may finally raise the question how the here suggested role of the resting state in objective-subjective transformation stands to the above proposed relational coding. I would argue that both are well compatible. While above I focused on the relation between intero- and exteroceptive stimuli, I now extend the focus to include the brain’s intrinsic activity and thus its neural stimuli into the equation of relational coding.

Let me be more specific. The incoming stimulus must be coded in relation to the intrinsic activity level and thus relative to it. The resulting neural activity must then be considered the integral of their interaction, i.e., rest-stimulus interaction, rather than being related to the stimulus alone. That though is possible only if neural activity is coded in terms of a relation between stimulus and intrinsic activity as distinguished from neural coding of the stimulus by itself. I thus assume rest-stimulus and stimulus-rest interaction to presuppose relational coding in very much the same way as the relation between intero- and exteroceptive stimuli is encoded into neural activity (see Northoff, 2012a for more details on the question of neural coding).

### “Non-affective-affective transformation”

How about the second feature of affective assignment, that non-affective-affective transformation? What functional mechanisms correspond to the transformation of a non-affective stimulus into an affective one? Panksepp (2010, p. 13) himself gives one hint in this direction. He considers primary process affect to be intrinsically valuative (in a wider sense as not being restricted to reward) in that it mirrors the value of environmental, bodily, and neural information for the organism. How can such value be generated, and what kind of functional mechanisms are necessary in order to value stimuli of different origin, exteroceptive, interoceptive, or neural?

In order for stimuli of various origins to be valued for the organism, they must be related to the organism itself, including its body and brain. More specifically, exteroceptive stimuli from the environment need to be related to the brain’s neural stimuli leading to rest-extero interaction and the body’s interoceptive stimuli leading to intero-extero interaction. The same holds for interoceptive stimuli which need to be related to the brain’s neural stimuli thus requiring rest-intero interaction. Finally, as demonstrated above, the brain’s resting state activity itself may be valued when rest–rest interaction occur.

How does affect enter these various interactions? Russell tells us that Core Affect is continuously present even in the absence of exteroceptive stimuli. Unlike exteroceptive stimuli which arise more episodically, there is continuous interoceptive input and thus continuous rest-intero interaction in the brain. Due to the continuous presence of the body, continuous interoceptive input and subsequent continuous rest-intero interaction cannot be avoided.

One may consequently consider rest-intero interaction as one possible candidate functional mechanism that may correspond to what Russell describes as Core Affect. It is by the continuous neural processing of the body’s interoceptive stimuli against the brain’s neural stimuli that affect may be generated. Hence, interaction of the interoceptive stimuli with the neural stimuli may transform the originally non-affective interoceptive stimulus into an affective one. The hypothesis is thus that rest-intero interaction may correspond on the functional level to the non-affective-affective transformation and thus to what Russell described as Core Affect.

However, there is not only Core Affect but also emotional episodes (Russell) or sensory affect (Panksepp) in relation to exteroceptive stimuli. How can exteroceptive stimuli be assigned affect and how can they undergo the non-affective-affective transformation? Very simple. They may be linked to interoceptive stimuli resulting in an intero-extero interaction. They would thereby be valued, which in turn would lead to a non-affective-affective transformation with the subsequent assignment of affect. Hence, one may consider the interaction of stimuli of various origins with specifically interoceptive stimuli from the body as a necessary condition for the non-affective-affective transformation. This may apply to the brain’s neural stimuli with rest-intero interaction which then leads to what Russell described as Core Affect and Panksepp as homeostatic affect. It may also apply to exteroceptive stimuli with intero-extero interaction that may then result in what Russell characterized as emotional episodes and Panksepp as sensory affect.

### Dissociation between subjectivity and affectivity

One may now be puzzled. I characterized objective-subjective transformation by the interaction of any kind of stimulus with the brain’s resting state activity, i.e., its neural stimuli, so that any kind of rest-stimulus interaction will do the job. And I considered the interaction of any stimulus with interoceptive stimuli from the body as being necessary for the non-affective-affective transformation. Hence, both transformations, objective-subjective and non-affective-affective are characterized by interactions with different stimuli, the brain’s neural stimuli and the body’s interoceptive stimuli.

As on a psychological level where affectivity and subjectivity co-occur, non-affective-affective, and objective-subjective transformations also co-occur in the “normal” case. There is interaction with the body’s interoceptive stimuli (e.g., intero-extero interaction), and there is interaction with the brain’s resting state and thus its neural stimuli (e.g., rest-intero and rest-extero interaction). This means that affectivity and subjectivity are co-constituted, which is reflected in both Panksepp and Russell definitions of affect by.

If one interaction takes over at the expense of the respective other, the co-constitution between affectivity and subjectivity may become dysbalanced. This is, for instance, the case in schizophrenia where rest-intero and rest-extero interactions may be reduced leading to an abnormal loss of subjectivity (Northoff and Qin, 2011). There is thus still non-affective-affective transformation while the objective-subjective transformation seems to fail: These patients thus still experience emotional feelings while their respective contents are no longer experienced as subjective but objective.

While the reverse seems to be the case in depression, where rest-intero interaction seems to predominate over intero-extero interaction (Alcaro et al., 2010; Northoff et al., 2011). In the most extreme case, depressed patients say that they no longer feel any emotion, the feeling of non-feeling. Hence, non-affective-affective transformation may be blocked while at the same time this state is experienced as highly subjective implying objective-subjective transformation. The cases of depression and schizophrenia thus indicate the possibility of dissociation between both forms of transformation.

Interoceptive processing and consecutively intero-extero interaction may also be altered or disrupted in alexithymia that concerns the inability to identify and describe emotional feeling. The exteroceptive stimuli and their respective contents can then no longer be associated with emotional feelings: While the contents are experienced as subjective (due to functioning rest-intero and rest-extero interactions), they are not assigned emotional feeling (due to deficient intero-extero interaction). Empirically this is supported by a recent study that shows the degree of interoceptive awareness to predict the degree of alexithymia with high interoceptive awareness going along with a low degree of alexithymia (Herbert et al., 2011).

## Conceptual Implications: Emotional Feeling and Consciousness

### Relational approach to emotional feeling

How should emotional feelings be conceptualized on the basis of the intero-exteroceptive relational model of neural coding? What we subjectively experience as emotional feeling is thus not so much mere perception of an interoceptive stimulus like the heartbeat perception but rather the relation between intero- and exteroceptive stimulus processing relative to our brain’s intrinsic activity. Emotional feelings can no longer be determined in an interoceptive-based way as perceptions of physiological body changes. Instead, emotional feelings may better be described in an neural-intero-exteroceptive relational way thus focusing more on the relation between brain, body and environment than on either the body or the environment itself^3^. What is constitutive of emotional feelings is thus the relation between brain, body, and environment so that feelings reflect the respective person’s relationship to the world.

This is paradigmatically reflected in what the philosopher Ratcliffe (2005, 2008) calls existential feelings. Based on Heidegger, he describes existential feelings as feelings that characterize our relation to the world, i.e., as ways of “finding ourselves in the world.” This is also pointed out by Solomon (2004, pp. 77–78, 84) in a more recent writing when he claims for “an existential notion of emotions” which he considers to be “subjective engagements within the world^4^.” For instance, different existential feelings characterize different relations to the world like feelings of homeliness, separation, belonging, power, control, etc. Most important, emotional feelings like anger, grief, etc. presuppose existential feelings so that both emotional and existential feelings can be characterized as relational. If so, the body itself may only be considered the medium through which feelings can be constituted. Feelings are the relation between person/body and environment rather than some perception of either bodily or environmental changes; in other terms, feelings are this relation implying that this relationship is felt.

Due to the very basic and foundational character of the brain-body-environment relation, the relational concept considers emotional (and existential) feelings basic and primary for emotions, i.e., feelings are then the “core nucleus” of emotions. This is very much in line with the neuroscientific approach by Panksepp (1998, 2005) who assumes what he calls “primary affective consciousness.” He regards “primary affective consciousness” as basic and crucial for all forms of subjective experience and thus for consciousness in general. Analogously, the relational view considers our relation to the world primary, basic and crucial to our subjective experience or, as Ratcliffe would probably say, the relation is existential.

### Neuro-phenomenal approach to emotional feeling

Since the relational concept characterizes the brain-body-environment as basic, primary, and constitutive of feelings, the here advanced relational concept of emotional feelings seems to complement the empirical approach by Panksepp in conceptual regard. Feelings and thus affective consciousness can only be primary and basic, as Panksepp claims, because they are our relation to the world. Another complementary point is Panksepp’s (and other authors like M. Sheets-Johnstone, N. Humphrey and R. Ellis) insistence on the close linkage between motor function and emotional feeling, i.e., the primary motor basis of affective consciousness.

In contrast to Damasio (2010), who opts for a rather sensory-based view of emotional feeling, Panksepp (and others like Ellis, 2005) argues for a primary “motor view” of affective consciousness and emotional feeling because all presumably involved subcortical regions like the PAG, the colliculi, etc. show strong connections to the motor system receiving motor afferences from and sending out motor efferences to other cortical and subcortical regions. Accordingly, Panksepp (and others like Ellis, 2005) claims that there is intrinsic linkage between motor action and emotional feeling resulting in what may be described as “I act, therefore I feel.” The assumption of motor underpinnings as being crucial to emotional feeling is well compatible with the relational concept.

The here suggested relational approach also needs to be distinguished from cognitive approaches. Cognitive approaches focus on the awareness and thus reflection of emotional and cognitive contents thus presupposing access or reflective consciousness, the awareness of subjective experience. For that various cognitive functions are assumed to be necessary. This is different in the relational approach that focus on phenomenal consciousness and thus on subjective experience itself and how it is generated and transformed into a phenomenal state (see below) on the basis of the brain’s neuronal states.

The relational approach can thus be characterized as “neuro-phenomenal approach” rather than “neuro-cognitive approach” (see Northoff, 2012b). Since the cognition, i.e., awareness and reflection, of subjective experience and its contents presupposes its generation, I assume the “neuro-phenomenal approach” to be more basic and prior to the neuro-cognitive approach. Future studies may therefore want to investigate how the here described neuronal processes of non-affective-affective and objective-subjective transformation impact cognitive functions and their respective neural substrates.

### Emotions and consciousness

**Figure 3:**
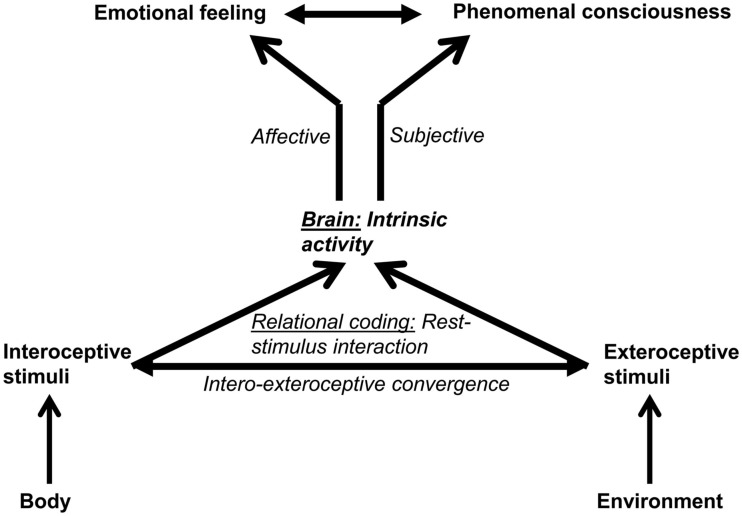
**The figure shows the relationship between emotional feeling and phenomenal consciousness**. By interacting with the brain’s intrinsic activity via relational coding affect and subjectivity is assigned to the intero- and exteroceptive stimuli. This in turn makes possible the generation of emotional feeling and phenomenal consciousness.

How is the transformation of the brain’s neuronal states into the phenomenal states of consciousness possible? The relational concept presupposes bilaterally dependent and constitutive linkage between brain, body, and environment. Mere linkage by sensory function would result in unilateral and rather instrumental linkage where the person/body cannot directly impact the environment. It is only by motor function that the person/body becomes intrinsically anchored in and non-instrumentally, i.e., constitutionally linked to the environment. In other terms, motor function must be considered the empirical means by means of which what I conceptually described as relational becomes possible. Panksepp’s insistence on motor underpinnings of emotional feelings may thus be considered complementary to the here advanced relational concept of emotional feeling (Figure 3).

Once emotional feelings are considered to be the core nucleus of both emotions and consciousness, the often made distinction between “having an emotion” and “feeling an emotion” becomes no longer applicable. Following Bennett and Hacker (2003, pp. 210–214), there is no principal distinction between “having an emotion” and “feeling an emotion” since, as Kripke (1972) already pointed out, the having of pain is to be identified with the feeling of pain. Either we have pain and subjectively experience or feel pain or we do not feel any pain and then we have no pain. “Having an emotion” is consequently to be identified with “feeling an emotion” and their distinction remaining untenable and implausible.

According to Bennett and Hacker (2003, p. 214), the main difference should better be drawn between “feeling an emotion,” as being identical with “having an emotion,” and “realizing what emotion one feels.” “Feeling an emotion” might then indicate subjective experience and thus what currently is called phenomenal consciousness (see below for further explication). In contrast, “realizing what emotion one feels” might be considered to implicate higher-order cognitive functions and thus be associated with what has been called reflective consciousness.

By considering feeling as constitutive of emotion and phenomenal consciousness, the relational concept of emotional feeling argues against the explanation of feelings in terms of higher-order cognitive and reflective functions mirroring what is called reflective consciousness. Roughly, reflective consciousness describes the person’s awareness that it has subjective experience and thus phenomenal consciousness – reflective consciousness may thus focus on higher-order cognitive functions.

Phenomenal consciousness, in contrast, does not describe cognitive and behavioral aspects associated with subjective experience. Instead, it focuses on the subjective-experiential aspect itself that is described as the “phenomenal aspect” (Chalmers, 1995; Block, 1996). A number of alternative terms and phrases pick out approximately the same core property of phenomenal consciousness. These include “qualia,” “phenomenology,” “subjective experience,” and “what it is like” which, despite subtle differences, we here consider to describe the same phenomenon for pragmatic purposes. I characterize emotional feeling by “qualia” and “what it is like” thus presupposing phenomenal consciousness.

The here proposed relational account is well in accordance with Peter Goldie’s approach who emphasizes the phenomenal, e.g., unreflective, qualitative, and “what it is like” character of emotional feeling (Goldie, 2000, pp. 68–69). Goldie (2000, pp. 1–2, 41) argues that the phenomenal character of feelings is due to the involvement of a point of view, a perspective, by means of which they become “fundamentally personal.” The relational concept claims that such personal point of view is established by constituting the relationship between brain, body, and environment and thus by constituting feelings be they existential or emotional. How such personal point can be established by relating brain and body to the environment remains to be discussed in detail which however is beyond the scope of this paper (see Northoff, 2004, 2012b; Northoff and Bermpohl, 2004; Northoff et al., 2006).

Finally, the here proposed neuronal mechanisms underlying especially the subjective nature of emotions may be relevant for consciousness in general. The yet to be specified and defined neuronal mechanisms underlying rest-stimulus interaction are assumed to be central for the subjective component. If so they must be regarded necessary of consciousness in general that is essentially defined as subjective. Even if not sufficient by themselves as neural correlates of consciousness (NCC), specific yet unknown ways of rest-stimulus interaction must then be regarded a necessary or predisposing condition of consciousness. One may consequently want to speak of neural predispositions of consciousness (NPC) as distinguished from the NCC (see Northoff, 2012b).

## Conclusion

The often favored James–Lange theory and many current neuroscientific approaches that consider feeling as mere perception of bodily changes and thus as “embodied” may be extended by considering the crucial role of the environment in directly constituting emotional feelings. I therefore suggested in this paper to complement the embodied concept of emotional feelings by a relational concept that assumes emotional feelings to be constituted by the brain-body-environment relationship. The relational concept assumes that the environment and the brain itself have not only instrumental and thus indirect impact on emotional feelings via the body but also a direct, e.g., non-instrumental and thus constitutional role in constituting emotional feelings.

The present paper focuses on whether such relational concept of emotional feelings is compatible with current empirical data on the neuroscience of emotion processing. If the relational concept of emotional feeling is empirically plausible, even interoceptive awareness should implicate brain regions that process exteroceptive stimuli. Both, e.g., intero- and exteroceptive brain regions, should then also be closely linked to each other in terms of anatomical, i.e., structural and functional connectivity.

Human brain imaging data show strong involvement of the VMPFC and other aCMS in emotional feelings. These regions can be characterized by strong convergence between intero- and exteroceptive inputs as well as of both with the brain’s high intrinsic activity, its resting state activity. This presupposes what I describe as the neural-intero-exteroceptive relational mode of neural coding rather than interoceptive-based translational neural coding (see also Northoff, 2012a). In short I assume relational coding to be the predominant neural code that allows to link emotions to brain, body, and environment. Emotions and emotional feelings may then be considered, metaphorically speaking, the bridge or glue between brain, body, and environment.

The intrinsic linkage between brain, body, and environment accounts for the subjective and affective nature of emotional feelings. By being processed in the brain relative to its intrinsic activity (at least in the human brain as it is designed) emotions cannot avoid becoming subjective and affective. The subjective and affective components must therefore be regarded intrinsic to and thus defining features of emotions. As such emotions and emotional feeling may be considered paradigmatic cases of consciousness in general which in the current neuroscientific and philosophical debate is rather often neglected (see also Northoff, 2012b).

## Conflict of Interest Statement

The author declares that the research was conducted in the absence of any commercial or financial relationships that could be construed as a potential conflict of interest.

## Footnotes

^1^It should also be pointed out that feelings cannot be considered to be conscious perceptions of the neural activity in those brain regions that induce emotion as for instance LeDoux assumes. We cannot become conscious of neural activity in the first-order emotion regions (see also Bennett and Hacker, 2003, 208) since we remain principally unable to perceive our brain’s neural activity as such which I recently called “autoepistemic limitation” (Northoff, 2004; Northoff and Musholt, 2006, see chapter 1 and 2 in Northoff, 2011).

^2^The here advanced relational concept may be considered an extension of the embodied approach by Colombetti and Thompson, who also emphasize the situated, extended and thus embedded nature of emotional feeling. Since the main focus here is on the neurophilosophical aspect, I cannot go into the philosophical details about the relational approach (see below for the discussion of some philosophical implications and Northoff, 2004 for a general outline). See also Ben-Ze’ev (1993, 81–99) who advocates a relational approach to perception and, in some part, also to emotion (see Ben-Ze’ev, 2000)

^3^This is well compatible with the relational approach to meaning and personal significance as suggested by Ben-Ze’ev (1993, 2000) that undercuts the traditional assumption that higher-order cognitive functions are necessary to give meaning and personal significance to otherwise meaningless and personally insignificant sense data.

^4^One may off course argue that we can have subjective experience without emotion in for instance so-called “cold” cognitions. “Cold” cognitions may however be considered just as an extreme case on a continuum in the relationship between emotion and cognition where feelings may still be involved in the background though being maximally suppressed.

## Appendix

### Glossarium

Coding: Formal measure according to which stimuli are encoded into neural activity.

Consciousness: Subjective experience characterized by “what it is like” and a point of view. Referring here mainly to phenomenal consciousness as distinguished from access or reflective consciousness.

Embeddedness: Constitutional (as distinguished from mere modulatory) dependence of consciousness and emotional feeling on the (social) environment.

Embodiment: Constitutional (as distinguished from mere modulatory) dependence of consciousness and emotional feeling on the body and its sensorimotor functions.

Exteroceptive stimuli: Input into the brain from the environment.

Interoceptive stimuli: Input into the brain from the own body.

Neural stimuli: Input into the brain from other regions and time points within the brain itself.

Non-affective-affective transformation: Processes that underlie the assignment of affect to a primarily non-affective stimulus that thereby becomes transformed into an affective stimulus.

Objective-subjective transformation: Processes that underlie the assignment of subjectivity to a primarily objective stimulus that thereby becomes transformed into a subjective stimulus.

Relational coding: Coding of different stimuli in relation to each other into neural activity in the brain.

Resting state: The state of the brain in the absence of any specific stimulus from outside the brain as from the body or the environment.

Rest–rest interaction: Changes in neural activity in the resting state. These changes may occur between different regions and/or across time as fluctuations in the spontaneous activity of the brain.

Rest-extero interaction: Interaction of the brain’s resting state activity with exteroceptive stimuli from the environment.

Rest-intero interaction: Interaction of the brain’s resting state activity with interoceptive stimuli from the own body.

Rest-stimulus interaction: Term for the interaction of interoceptive and/or exteroceptive stimuli with the brain’s resting state activity.

Translational coding: Coding of each stimulus by itself into neural activity in the brain with subsequent translation of the different neural activities into each other.
